# Identification of Signature Genes and Characterizations of Tumor Immune Microenvironment and Tumor Purity in Lung Adenocarcinoma Based on Machine Learning

**DOI:** 10.3389/fmed.2022.843749

**Published:** 2022-02-25

**Authors:** Haiming Feng, Ye Zhao, Weijian Yan, Xiaoping Wei, Junping Lin, Peng Jiang, Cheng Wang, Bin Li

**Affiliations:** ^1^Department of Thoracic Surgery, Second Clinical Medical College, Lanzhou University, Lanzhou, China; ^2^First Clinical Medical College, Lanzhou University, Lanzhou, China

**Keywords:** lung adenocarcinoma, machine learning, tumor immune microenvironment, tumor purity, gene expression, signature genes

## Abstract

The implication of the Estimation of Stromal and Immune cells in Malignant tumor tissues using expression data (ESTIMATE) method to determine the tumor microenvironment (TME) and tumor immune score including tumor purity represents an efficient method to identify and assess biomarkers for immunotherapy response in precision medicine. In this study we utilized a machine learning algorithm to analyze the Cancer Genome Atlas (TCGA) and Gene Expression Omnibus database (GEO) lung adenocarcinoma (LUAD) transcriptome data to evaluate the association between TME and tumor purity. Furthermore, we investigated whether fewer TME components or a few dominant genes can infer tumor purity. The results indicated that the 29 immune infiltrating components determined by the ssGSEA method could screen the 5 TME components [chemokine C-C-Motif receptor (CCR), T-helper-cells, Check-point, Treg, and tumor-infiltrating lymphocytes (TIL)] that significantly contributed the most to tumor purity prediction through regression tree and random forest regression methods. The findings revealed that higher activity of these five immune infiltrating components significantly lowered the tumor purity. Moreover, 5 TME components contributed significantly to the improvement of Mean Square Error (MES); therefore, we selected these five sets' genes and analyzed survival data to establish a prognostic model. We screened out 11 prognostic-related genes and constructed a risk model comprising 11 genes with good predictive value for patients' prognosis. Furthermore, we obtained four genes (GIMAP6, CD80, IL16, and CCR2) that had predictive advantages for tumor purity using random forest classification and random forest regression. The comprehensive score of genes for tumor purity prediction (CSGTPP) was obtained by least absolute shrinkage and selection operator (LASSO) regression indicated that four genes could be successfully used to classify high and low CSGTPP samples and that tumor purity was negatively correlated with CSGTPP. Survival analysis revealed that the higher the CSGTPP, the better the prognosis of patients. The association between a cluster of differentiation 274 (CD274) and CSGTPP revealed a higher expression of CD274 in the high CSGTPP group. Collectively, we speculated that CSGTPP could serve as a predictor of the response to immunotherapy and a promising indicator of immunotherapy effect.

## Introduction

The tumor microenvironment (TME) represents a dynamic cellular milieu consisting of tumor cells, extracellular matrix (ECM), the blood and lymphatic vasculature, stroma, fibroblasts, infiltrating immune cells, and neighboring tumor related non-tumor cells. The immune system plays a critical role in immunosurveillance, as the immune cells of the immune system can recognize and eliminate tumor cells within the TME, thereby contributing to tumor progression. However, to evade the immune surveillance, tumor cells adopt multiple strategies to avoid immune recognition and instigate an immunosuppressive TME. Accumulating evidence indicates that defects in any of these mechanisms might contribute to the failure of the anti-tumor immunity and immune escape (1). Different tumor types adopt different strategies to escape the immune surveillance and killing of tumor cells by the immune system, thereby generating immune tolerance and promoting tumor occurrence and development (2). Immunotherapy, aiming to restore and boost the body's natural defenses to eradicate tumor cells, has emerged as a breakthrough therapeutic strategy for cancer. Several classes have emerged within immunotherapeutic agents, including monoclonal antibody immune checkpoint inhibitors, therapeutic antibodies, cancer vaccines, cell therapy, and small molecule inhibitors (3). In particular, the immune checkpoint inhibitors have shown potent anti-tumor activity across multiple malignancies such as melanoma, non-small cell lung cancer, kidney cancer, and prostate cancer. Several immunotherapy drugs have been approved by the US FDA (Food and Drug Administration, FDA) for clinical application (4). Immunotherapy can produce a more significant sustained response in patients with advanced cancer than conventional chemotherapy (5). However, this response only occurs in a small subset of patients. The efficacy of immunotherapy usually depends on the infiltration of immune cells into the TME. The immune cells of immune systems infiltrate into the TME and contribute to the modulation of tumor progression. TME is highly heterogeneous and consists of tumor cells, stromal cells, ECM, and immune cells that drive tumor cells fate to progression and metastasis. The immune system *in vitro* can recognize tumor antigens and kill tumor cells. Increasing studies have highlighted the complex and dynamic interactions between cells of the immune system and the TME in cancer progression. Moreover, TME plays an essential role in suppressing or enhancing the immune response. Understanding the complexity of interactions between TME and the immune cells will help select patients for immunotherapy and improve the curative effect of patients with immunotherapy. Tumor purity represents the proportion of tumor cells in tumor tissue (6). Studies have shown that tumor purity is significantly associated with the clinical characteristics, genome expression, tumor's biological characteristics, and prognosis of patients with cancer. It is noteworthy that ignoring the impact of tumor purity can lead to systematic bias in tumor genomic analyses, recurrence risk, and efficacy prediction. An accurate assessment of tumor purity is helpful to analyze tumor samples objectively. Therefore, the present study aimed to investigate the relationship between the immune microenvironment and Lung adenocarcinoma (LUAD) tumor purity through machine learning algorithms. And explored if tumor purity can be inferred from the genomic analyses, and investigated the correlation between tumor purity and immunotherapy.

## Materials and Methods

### Data Source and Pre-processing

The RNA-Seq based transcriptome profiles (FPKM; Fragments per Kilobase of transcript per Million mapped reads) and corresponding clinical data of LUAD patients were downloaded from the TCGA portal, the gdc-client software download tool. Additionally, the expression profiles of LUAD patients (GSE68465, sequenced using Affymetrix, HG-U133A plus 2.0 Array, up to November 2020) were also obtained from the GEO database (http://www.ncbi.nlm.nih.gov/geo/). All analyses were performed using R software (R Foundation for Statistical Computing, Vienna, Austria, 3.4.1 Version).

### Assessment of the Degree of Tumor Immune Infiltration

Using the most well-recognized and commonly used immune cell marker genes (7), we assessed the infiltration of different types of immune cells by single-sample Gene Set Enrichment Analysis (ssGSEA) with Gene Set Variation Analysis (GSVA) package in R package. Subsequently, based on the ssGSEA value, the samples were divided into high, medium, and low immune activity clusters.

### Calculation of the Immune Score of the TME

We obtained stromal score, immune score, estimate score, and tumor purity based on the transformed expression matrix, and tumor purity was calculated through the “Estimate” R package (8).

### Determination of the Primary Immune Infiltration Gene Sets Using Machine Learning

Using regression trees and random forest regression, we established a regression model and assumed that immune infiltration and immune purity were correlated. Then, based on the ssGSEA sets, we selected the ssGSEA sets that most significantly contributed to the improvement in Mean Square Error (MES) with “partykit” and caret package in R. TCGA data were randomly divided into training set and validation set with the ratio of 7:3, GSE68465 data were used as the test set.

### Correlation Between Target ssGSEA Sets and Tumor Purity

The association between the target ssGSEA sets value and tumor purity in high, medium, and low immunocompetent samples was determined using the “ggally” package in R.

### Survival Analysis of Target ssGSEA Sets and Tumor Purity

According to the target, ssGSEA sets value and tumor purity; the samples were divided into high and low groups. We further investigated the clinical data and survival outcome (excluding samples with missing clinical data and survival time of fewer than 30 days) to assess the association between each clinical characteristic and prognosis with the “survminer” package in R software.

### LASSO Regression Analysis for the Construction of the Prognostic Gene Model

Univariate Cox proportional hazards regression analysis was performed to screen target ssGSEA sets genes significantly associated with overall survival (OS) in the TCGA LUAD dataset. Then, using R-glmnet package, we performed LASSO Cox regression analysis of the identified OS-related genes. Multivariable Cox proportional hazards regression analysis was performed to establish the prognosis model of target ssGSEA sets genes. The LUAD samples were divided into high risk and low risk by the median risk score; the Kaplan–Meier curve was constructed, and the log-rank test was conducted to compare the survival differences between the two groups. The ROC curve was used to evaluate the accuracy of the model. GSE68465 data was utilized as the validation set to further evaluate the model.

### The Implication of Target Genes for Tumor Purity Classification

We established the corresponding relationship between the sample gene expression matrix and the tumor purity after screening the target gene using Cox proportional hazards regression analysis. Using the random forest regression method R-random Forest package, we constructed the model to evaluate the linear relationship between the target genes and the tumor purity and screen the most significantly contributed genes to the improvement in MES value. Then, we divided the samples into two groups according to tumor purity. Subsequently, a relationship with gene expression data was analyzed to evaluate the effect of the target genes' classification on tumor purity by random forest classification method achieved by R-random Forest package. At the same time, the average reduction of the Gini index was used to evaluate the contribution of the genes to the classification, and significantly associated genes were selected based on the method of random forest regression. TCGA data were divided into a training set and validation set at a ratio of 7:3; GSE68465 data was used as a test set.

### The Implication of Target Genes in the Sample Classification

In order to visualize the effect of the target genes on the sample classification, we used Principal Component Analysis (PCA) to classify the samples corresponding to the target gene by using the R packages “FactoMineR” and “factoextra.” In this step, we sorted the samples of TCGA and GEO according to tumor purity. The first 33% of samples presented low purity samples, and the last 33% of samples represented high purity samples.

### Lasso Regression Analysis of Target Gene to Predict the Effect of Immunotherapy

We constructed a LASSO regression model from the target genes screened in TCGA LUAD data and the tumor purity of the corresponding sample to calculate the comprehensive score of genes for tumor purity prediction (CSGTPP) = ∑ Xα^*^ coef α, where coef α is coefficient, and Xα is gene relative expression, the samples were divided into high CSGTPP and low groups. PCA was applied to analyze the classification effect of the target genes and the survival prognosis of the two groups. And then, we analyzed the differences in target genes and tumor purity between the two groups. Finally, we further explored immunotherapy's effect by comparing the differences in the immune target gene, CD274, in the two groups.

## Results

### ssGSEA Sets Value and Tumor Purity

We identified 535 TCGA LUAD gene expression matrices and 443 GEO gene expression matrices and analyzed the results of 29 immune infiltration sets in LUAD. Simultaneously, we determined tumor purity scores by the ESTIMATE method. We excluded samples with incomplete survival data and survival time <30 days after sorting out the clinical data. A total of 443 TCGA clinical survival samples and 442 GEO clinical samples were included to construct the LASSO regression model. However, we eliminated clinical samples with incomplete TNM data, and finally, 307 TCGA clinical samples and 339 GEO clinical samples were selected for subsequent analysis.

### Distribution of Immune Infiltrating Gene Sets and Tumor Purity in Different Immune Active Samples

**Figure 1:**
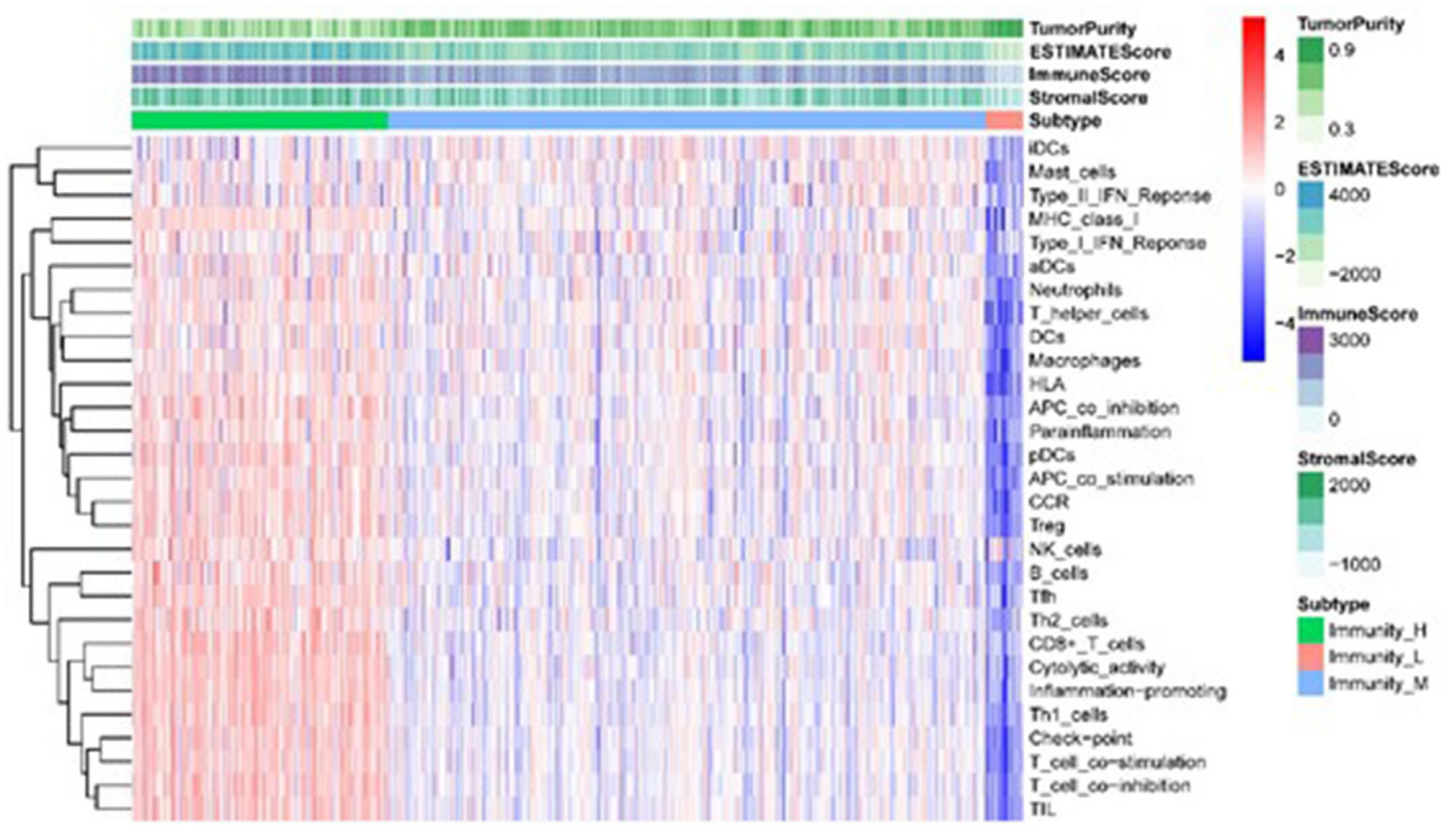
Heatmap of the correlation between TME sets and immune score in different immunologically active samples.

Using the 29 immune infiltration sets with the “sparcl” package in R, we calculated the immune activity of 535 TCGA and 443 GEO LUAD samples and divided them into three groups of low, medium, and high. Cluster analyses indicated the immune infiltration and immune scores of the three groups of tumor samples, and further analysis of the results revealed that with the increase in immune activity, 29 immune infiltrating gene sets and estimateScore were positively correlated. In contrast, tumor purity decreased with the increase in tumor immune activity (Figure 1).

### Predominant Immune Infiltration Gene Sets

**Figure 2:**
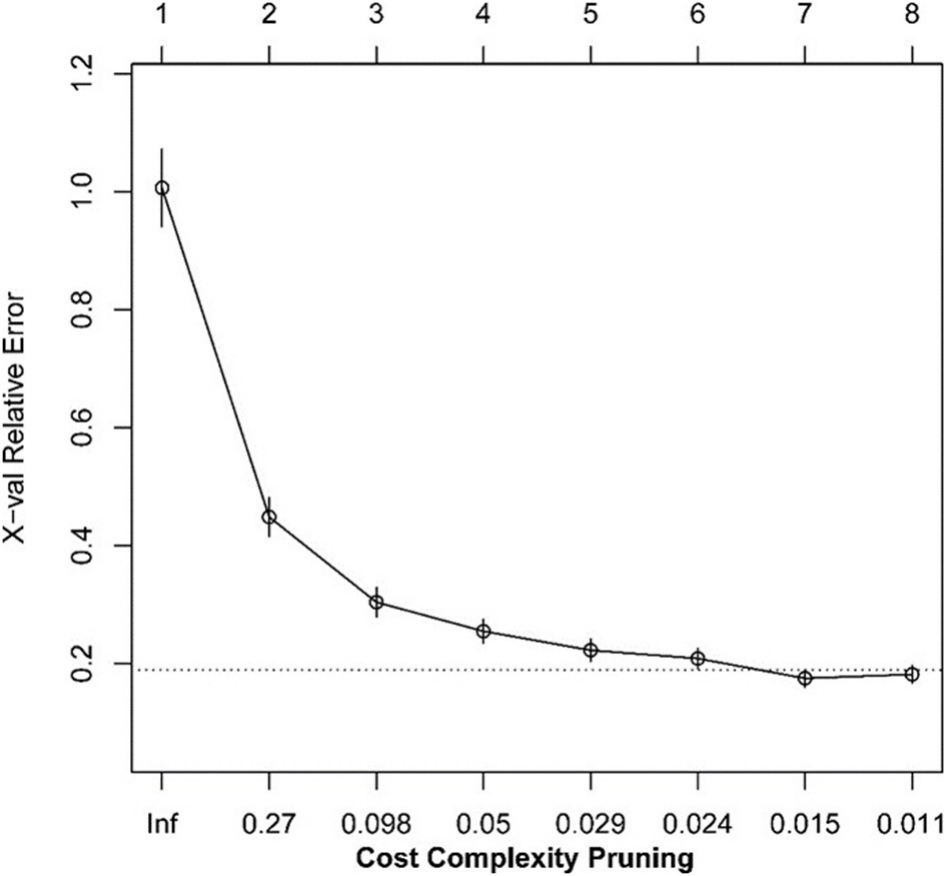
The relationship between the scale of the regression tree and the relative error.

**Figure 3:**
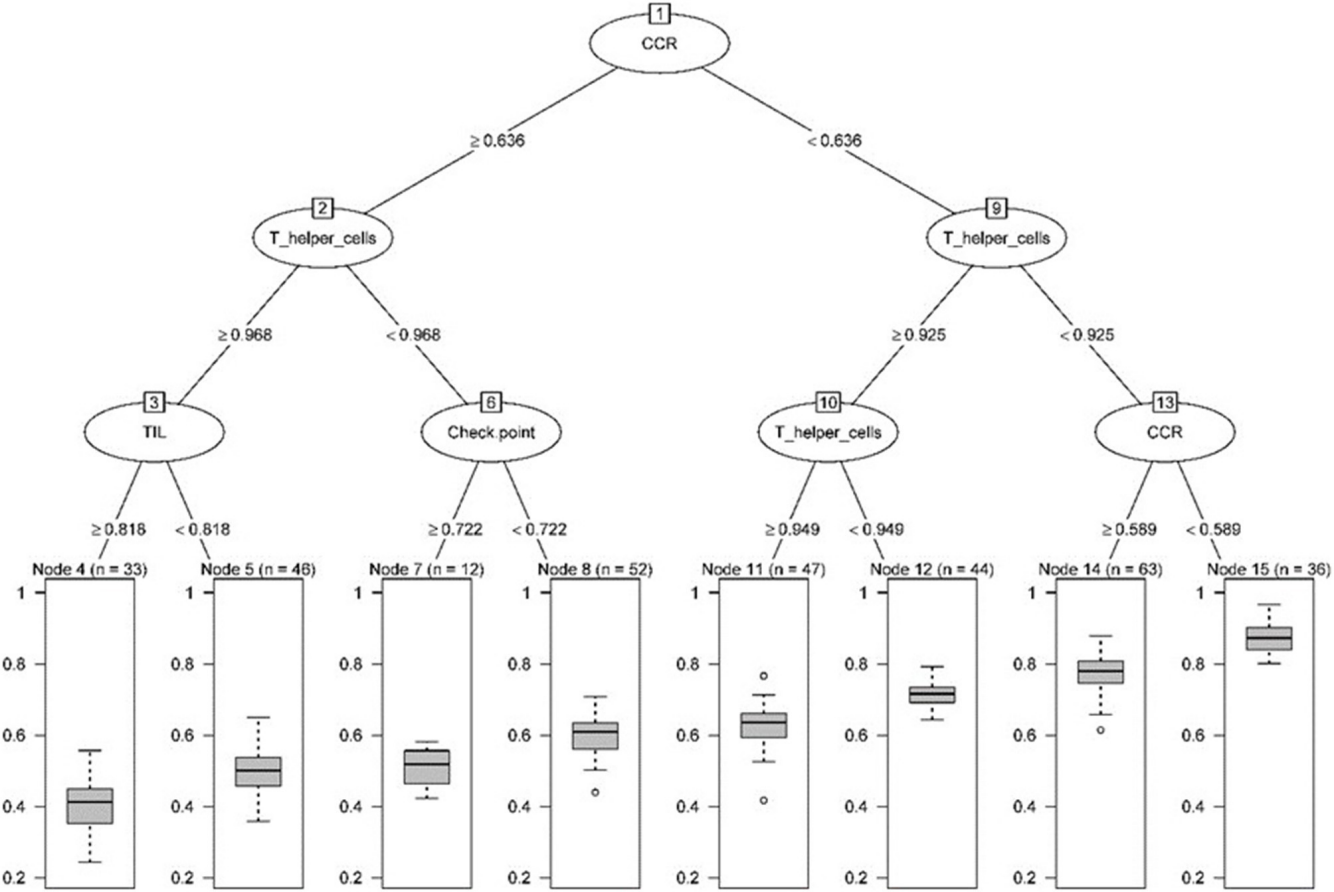
Pruning tree containing the number of splits, nodes, observation TME sets, and prediction results.

We transformed 29 immune infiltration sets and tumor purity into corresponding matrices, divided TCGA LUAD data into training set and validation set at a ratio of 7:3, and used regression tree to build the predictive model, GEO LUAD data was used as the test set to verify the model. MES is an excellent indicator of the calibration model. After constructing the model with the “Rpart” function and using 10-fold cross-validation, it was found that when the number of splits was 6, there was the smallest splitting error (xerror = 0.175; Figure 2); therefore, we chose the tree size to be 7, and used the “partykit” package to reduce branches, and apparently received 8 prediction results (Figure 3). Moreover, we used the “predict” function to envisage the validation set and the calculated MES to be 0.004, indicating that model was stable. Similarly, we used GEO data for testing, and the calculated MES value was 0.083. The model exhibited a good countermeasure effect.

**Figure 4:**
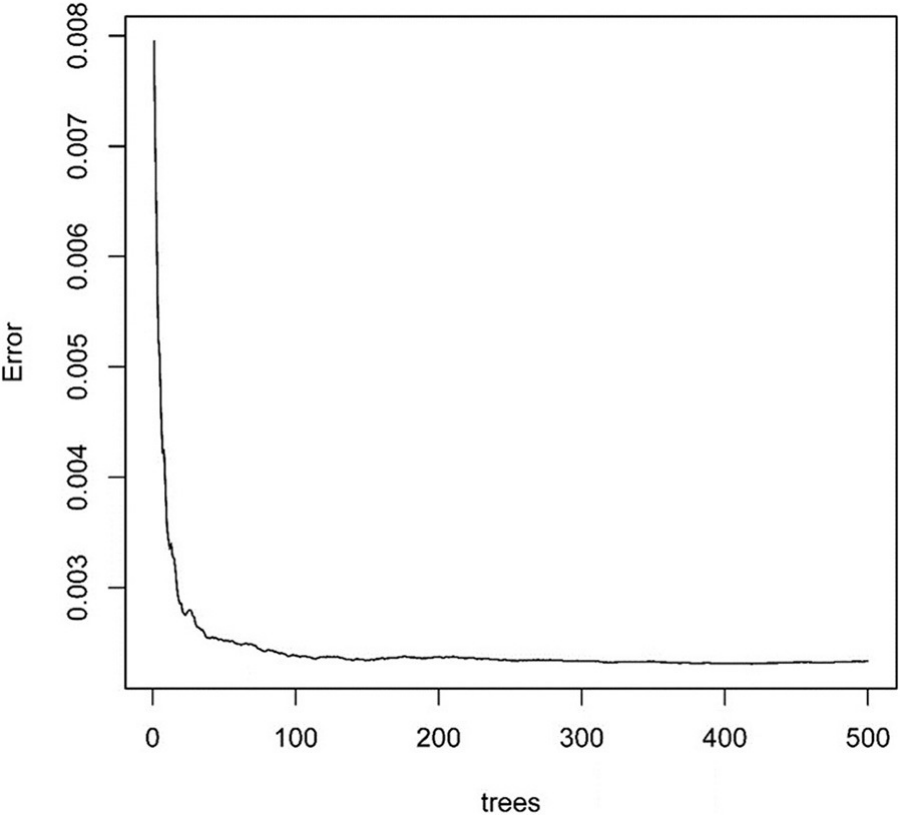
The relationship between split tree count and error.

**Figure 5:**
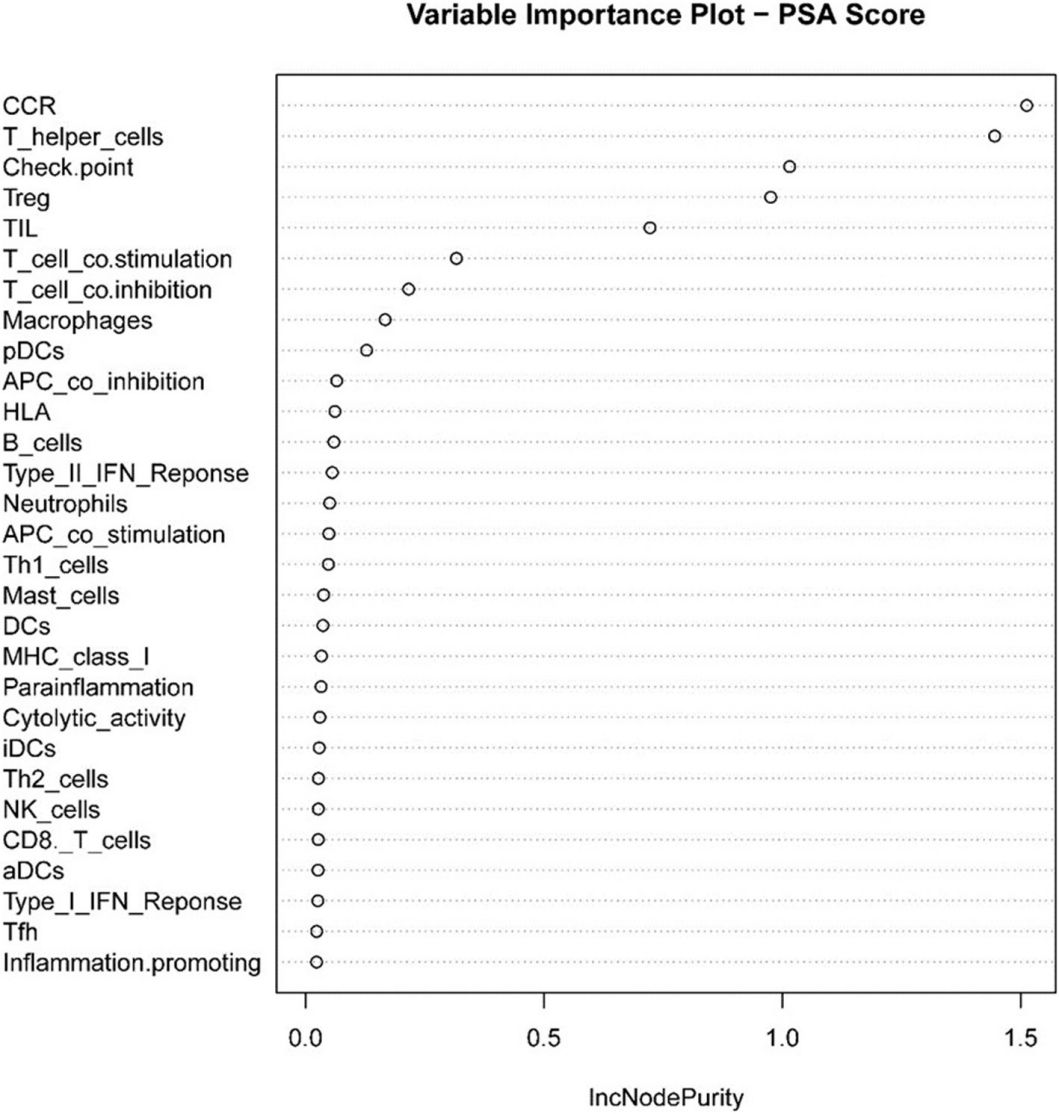
Statistical chart of important TME sets contributed to tumorpurity and MES improvement percentage (IncNodePurity).

Next, we used the “randomForest” function to perform regression analysis on the aforementioned data. We estimated that the number of specific optimal trees was 419, but from the relationship between the MES and the number of trees in the model, it could be observed that as the number of trees increases to about 100 trees, the error improvement was not evident, therefore, we chose 100 trees as the random forest regression (Figure 4). The resulting MES obtained was 0.002, and nearly 89.28% of the variance was explained. To end, we analyzed the critical scalar that drives the result. As represented in Figure 5, CCR, T-helper-cells, Check-point, Treg, and TIL contributed significantly to the improvement of MES. The model presented good MES values with the validation and test sets, which were found to be 0.002 and 0.087, respectively. On comparison of the two methods of regression tree and random forest regression, the results indicated that both models had good predictive value. Therefore, we selected CCR, T-helper-cells, Check-point, Treg, and TIL immune infiltration sets as our target sets.

### Association Between Target ssGSEA Sets and Tumor Purity

**Figure 6:**
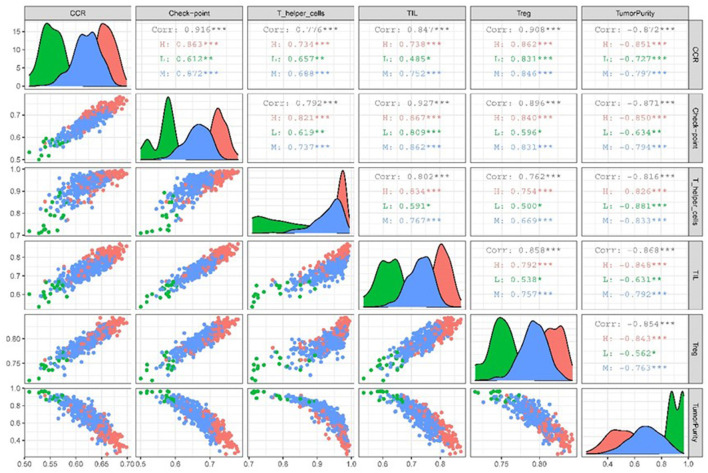
Scatter plot of correlation matrix between target TME sets and tumor purity in different immunocompetent samples.

We used the “Ggally” package in R software to estimate the correlation between target immune infiltration sets and tumor purity among different immunologically active samples from TCGA LUAD data. The results revealed that tumor purity and immune infiltration among different immunologically active samples were significantly negatively correlated (Figure 6).

### Survival Prognosis Analysis of Target ssGSEA Sets and Tumor Purity

**Figure 7:**
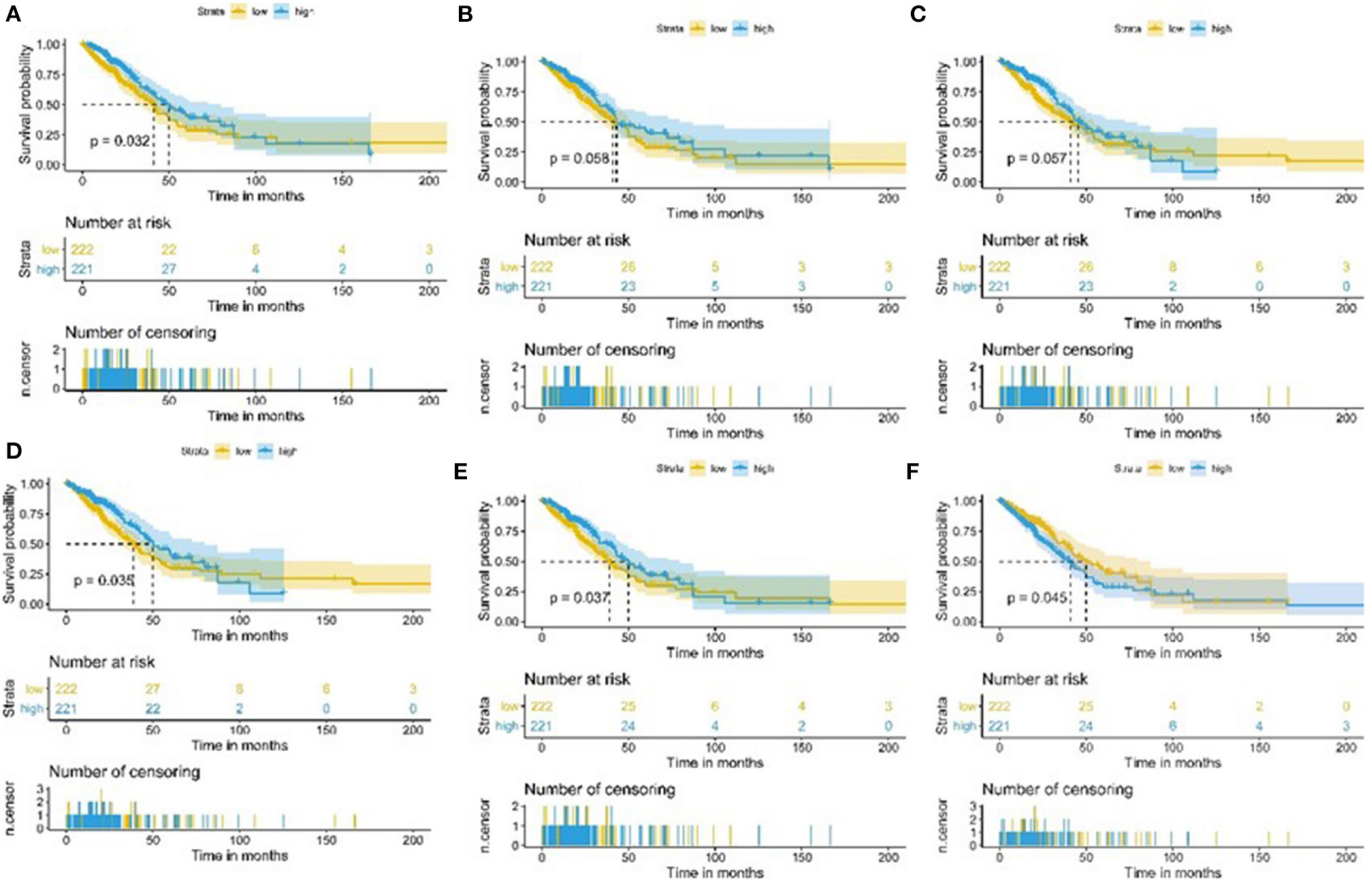
Kaplan-Meier curve of patients with high and low TME sets and tumor purity group. **(A)** Kaplan-Meier curve of patients with high and low CCR2 group; **(B)** Kaplan-Meier curve of patients with high and low T-helper-cells group; **(C)** Kaplan-Meier curve of patients with high and low Check-point group; **(D)** Kaplan-Meier curve of patients with high and low Treg group; **(E)** Kaplan-Meier curve of patients with high and low TIL group; **(F)** Kaplan-Meier curve of patients with high and low Tumorpurity group.

We used 443 TCGA LUAD samples to investigate further the relationship between target immune infiltration sets and tumor purity and survival prognosis; the results suggested that the lower the purity of the tumor, the better the prognosis. Conversely, it was also evident that the higher the degree of immune infiltration, the better the patient's survival (Figure 7).

### Prognostic Model and Genes Associated With Prognosis

**Figure 8:**
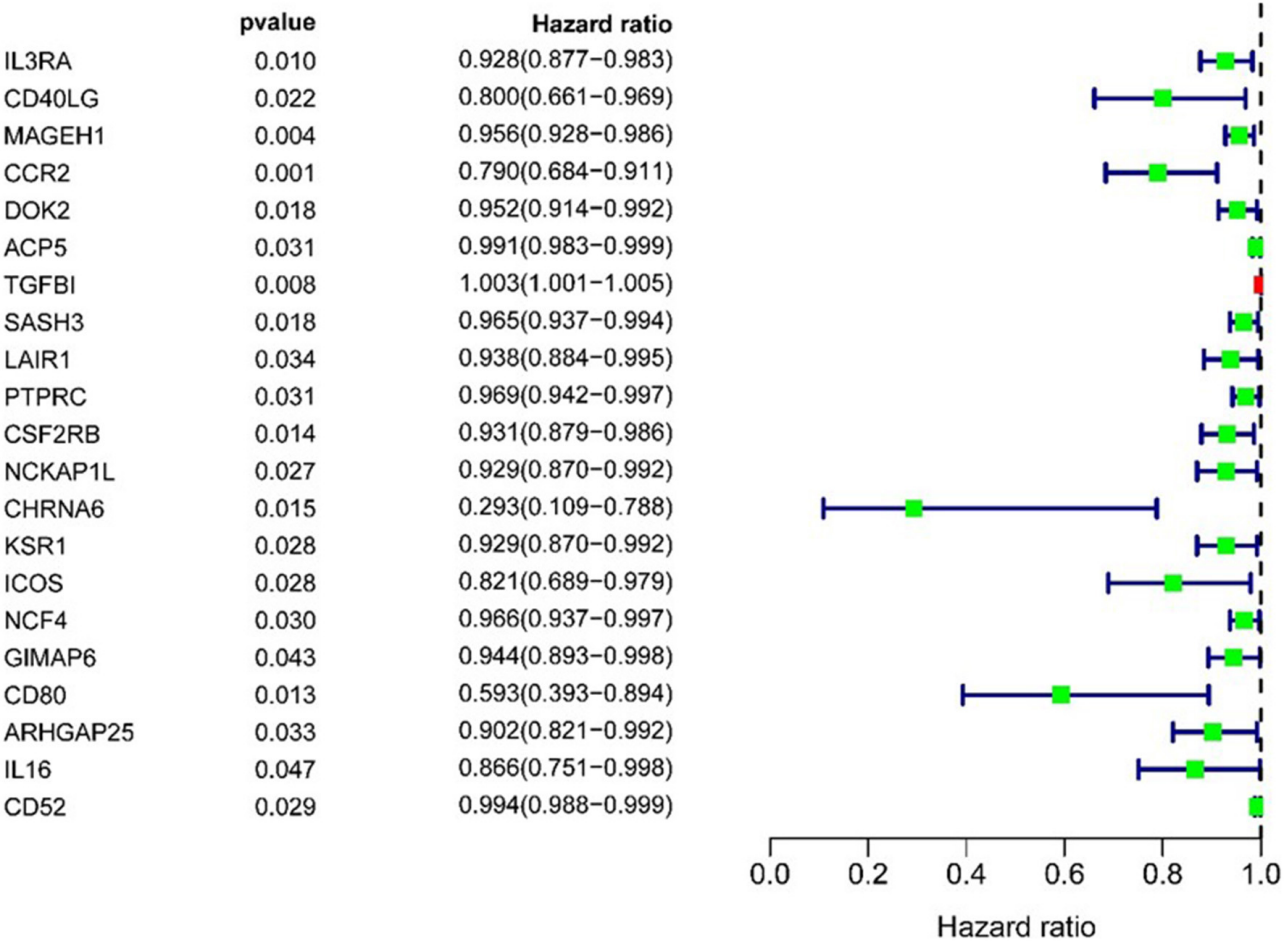
Target genes screened by univariate prognostic analysis.

One hundred and three immune infiltrating genes from CCR, T-helper-cells, Check-point, Treg, and TIL sets of TCGA LUAD data were analyzed by Univariate Cox regression. There were 21 genes associated with a prognosis and entered into the LASSO regression analysis (Figure 8); a total of 11 genes (IL3RA, MAGEH1, CCR2, ACP5, TGFBI, CHRNA6, KSR1, GIMAP6, CD80, IL16, and CD52) were identified for building model. The coefficients of each gene were presented in Table 1.

**Table 1:** The coefficients of genes for building model.

**Genes**	**Coefficients**
IL3RA	−0.0323529240412679
MAGEH1	−0.0313240392509636
CCR2	−0.258547384333454
ACP5	−0.00104506851750573
TGFBI	0.00291649350382021
CHRNA6	−0.473404652074516
KSR1	−0.0660881115987976
GIMAP6	0.020759981275271
CD80	−0.188951143790105
IL16	0.128342508954468
CD52	−0.000223112433304806

**Figure 9:**
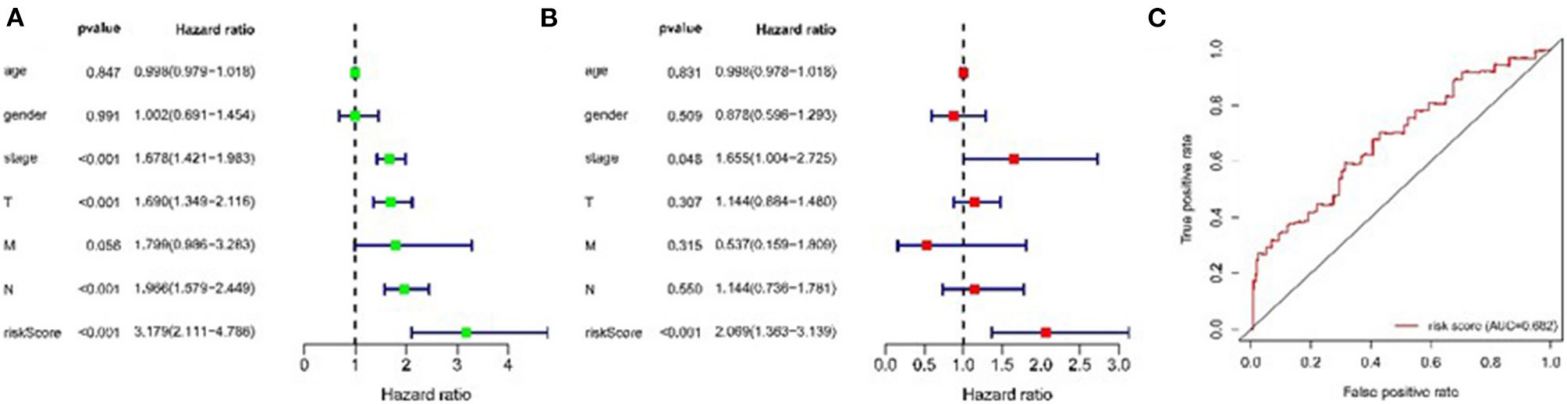
Construction of TME-related genes model for patients with LUAD. **(A)** Prognostic values of TME-related genes by univariate Cox regression analysis. **(B)** Prognostic values of TME-related genes by multivariate Cox regression analysis. **(C)** ROC curve of TME-related genes.

**Figure 10:**
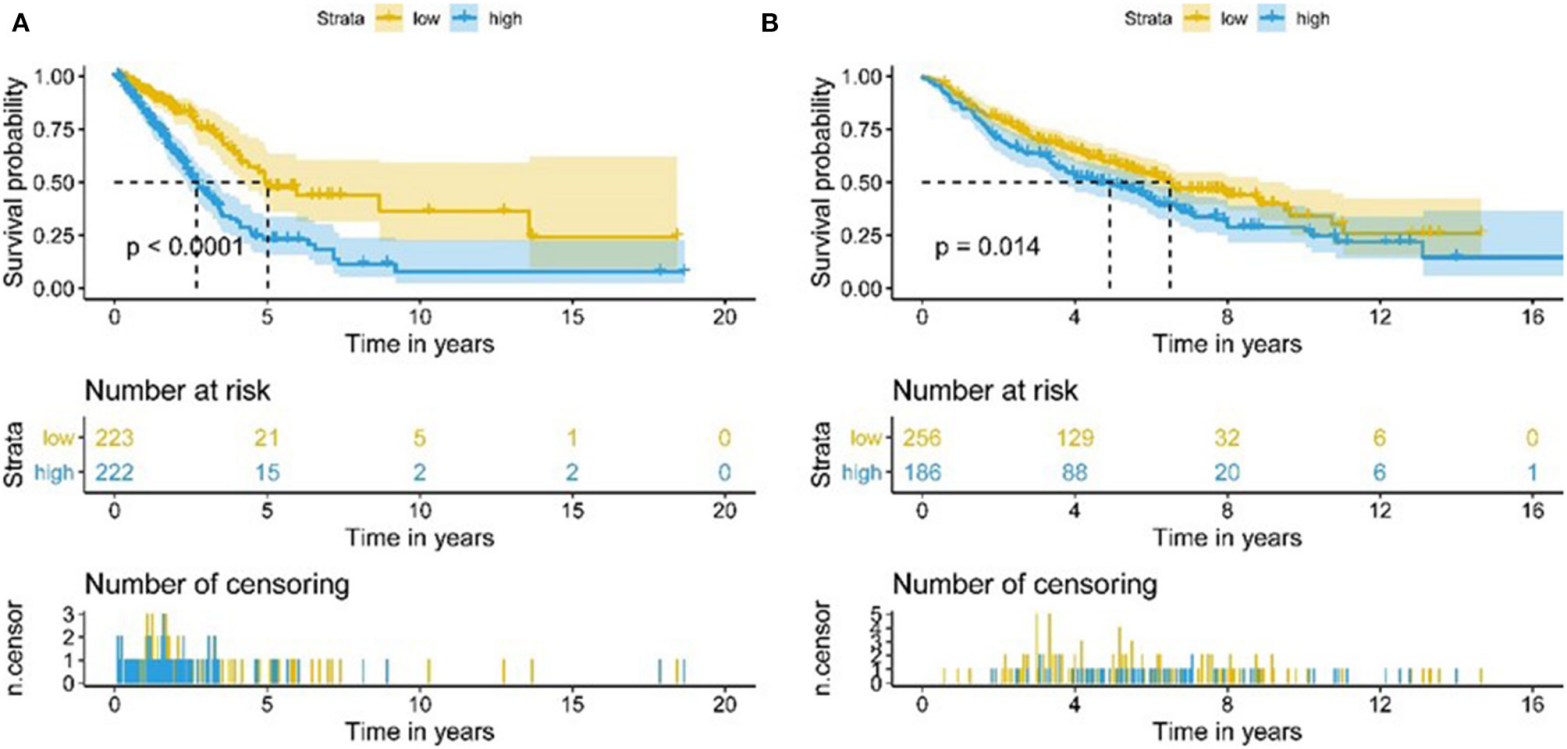
Kaplan-Meier analysis of OS for LUAD patients using TCGA and GEO database. **(A)** Kaplan-Meier survival curves of the relative OS of high- and low-risk groups in TCGA database. **(B)** Kaplan-Meier survival curves of the relative OS of high- and low-risk groups in GEO database.

We assessed the prognostic value of risk scores, which were estimated with the formula risk score = ∑ Xβ^*^ coef β, where coef β was coefficient, and Xβ was gene relative expression. For TCGA LUAD data, the risk score in both univariate and multivariate analysis was significantly related to overall survival (OS) (HR = 3.179, 95% CI = 2.111–4.786, *P* < 0.001; HR = 2.069, 95% CI = 1.363–3.139, *P* < 0.001, respectively.) (Figures 9A,B). It was observed that the patients with low-risk scores exhibited significantly better prognosis than those with a high-risk score (Figures 10A,B) both in TCGA and GEO LUAD data by the Kaplan–Meier cumulative curve. The AUC of risk score was 0.68, which implied that the Cox model could pretty well-predict the prognosis (Figure 9C).

### The Implication of Target Genes for Tumor Purity Classification

**Figure 11:**
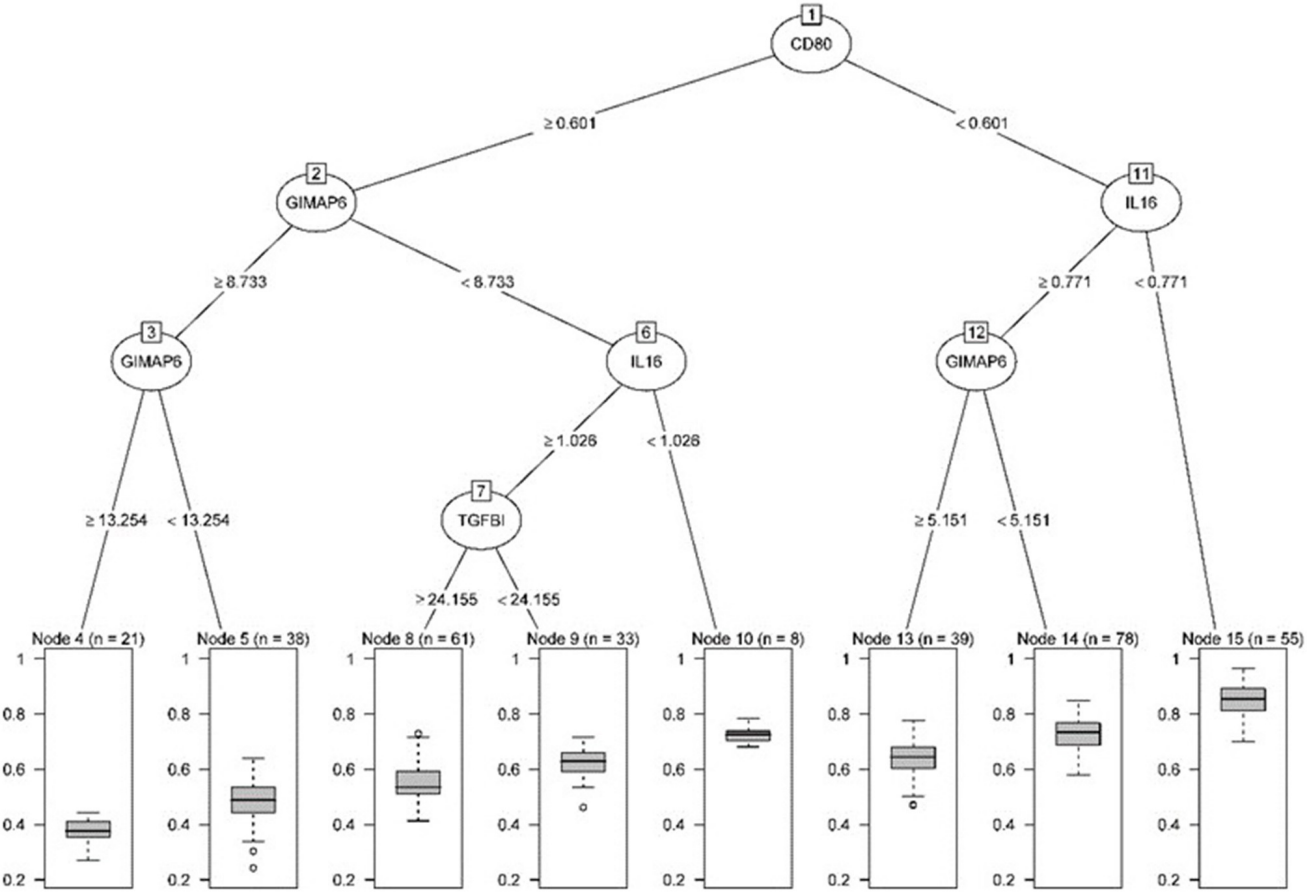
Pruning tree containing the number of splits, nodes, observation genes, and prediction results.

**Figure 12:**
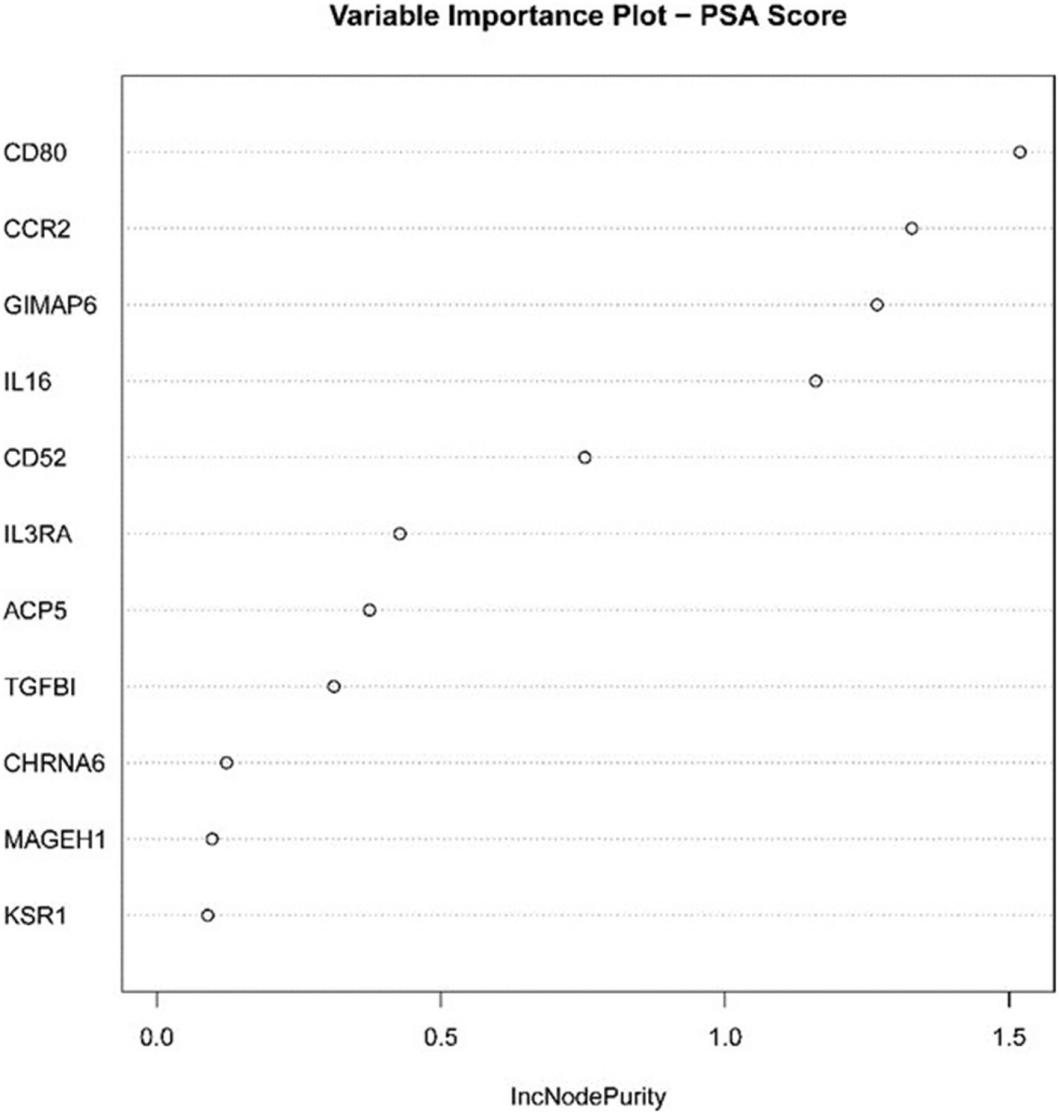
Statistical chart of important TME-related genes contributed to the model and MES improvement percentage (IncNodePurity).

We established the corresponding relationship between the 11 genes expression matrix and the tumor purity and performed random forest regression to predict the linear relationship between 11 gene expression and tumor purity. As mentioned before, 465 TCGA LUAD data were divided into a training set and validation set at a ratio of 7:3, while 443 GEO LUAD data was used as the test set to verify the model. We selected 100 trees for the random forest regression analysis, the resulting MES was 0.003, and almost 85.45% of the variance was explained. Finally, we obtained four genes (CD80, CCR2, GIMAP6, and IL16) that significantly improved MES. The model exhibited good MES values on the validation and test sets, which were 0.003 and 0.094, respectively (Figures 11, 12).

**Figure 13:**
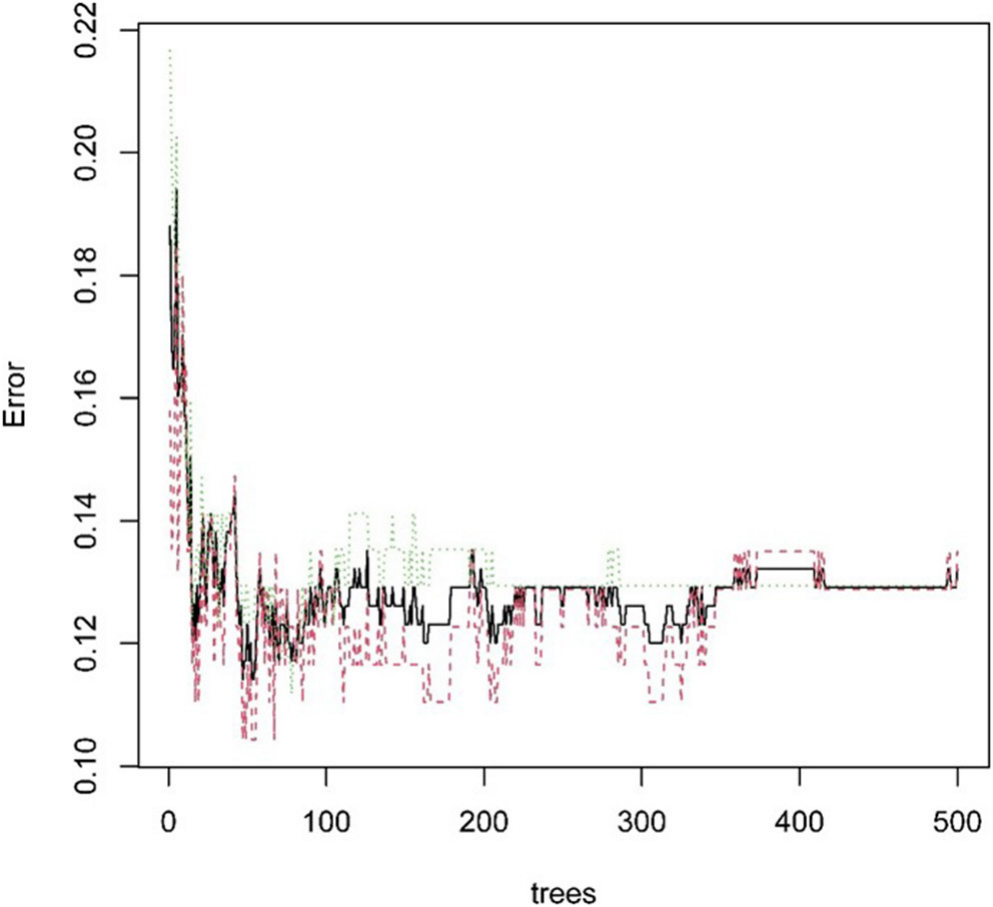
Statistical graph of the relationship between the prediction error and the number of split trees.

**Figure 14:**
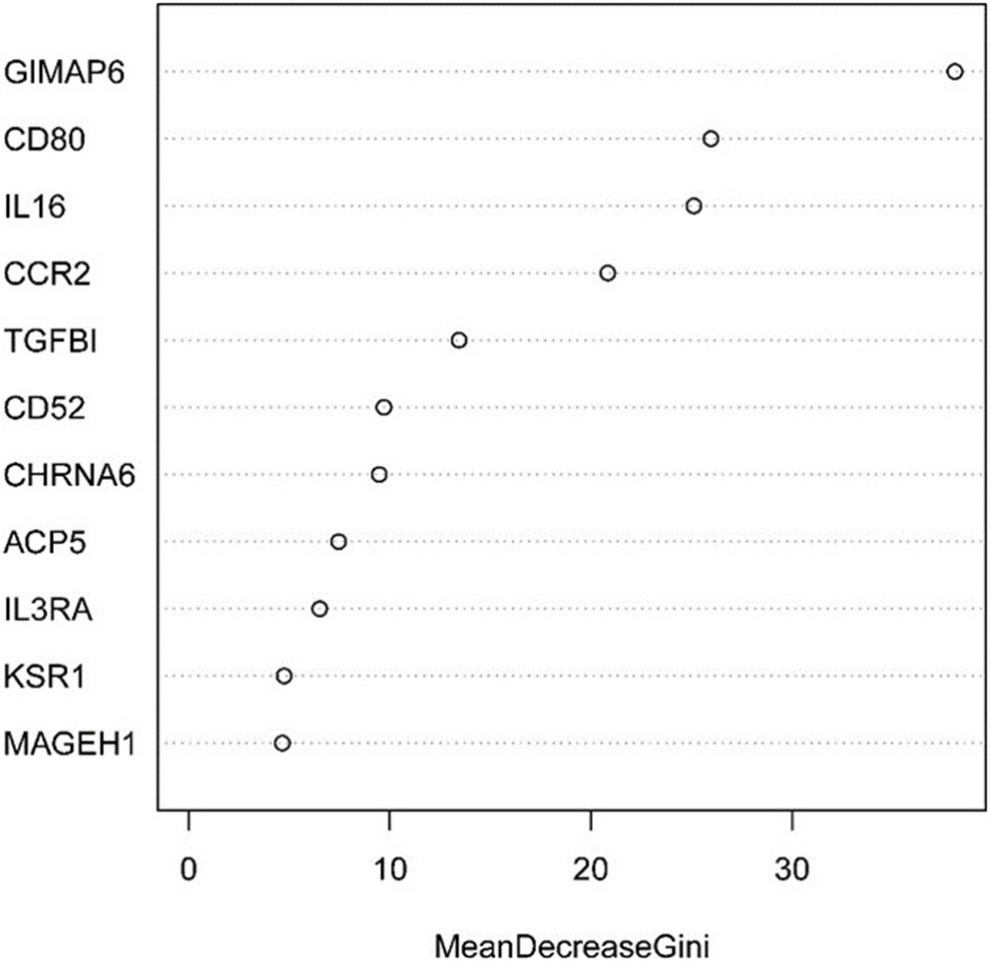
Statistical chart of important TME-related genes contributed to the model and Gini Index.

Next, the above data were classified by random forest, and the classification tree was selected as 47 trees for the model construction; therefore, the error rate obtained was 11.41% with an accuracy rate of 88.59% (accuracy = (146+149)/333). The accuracy of the prediction results on the validation set and test set was 90.91% (accuracy = (61+59)/132) and 97.41% (accuracy = (224+229)/465), respectively. Using the correlation between genes and Gini index, we obtained 4 most contributing genes (GIMAP6, CD80, IL16, and CCR2), which were consistent with the random forest regression for the target genes (Figures 13, 14).

### The Implication of Target Genes in the Sample Classification

**Figure 15:**
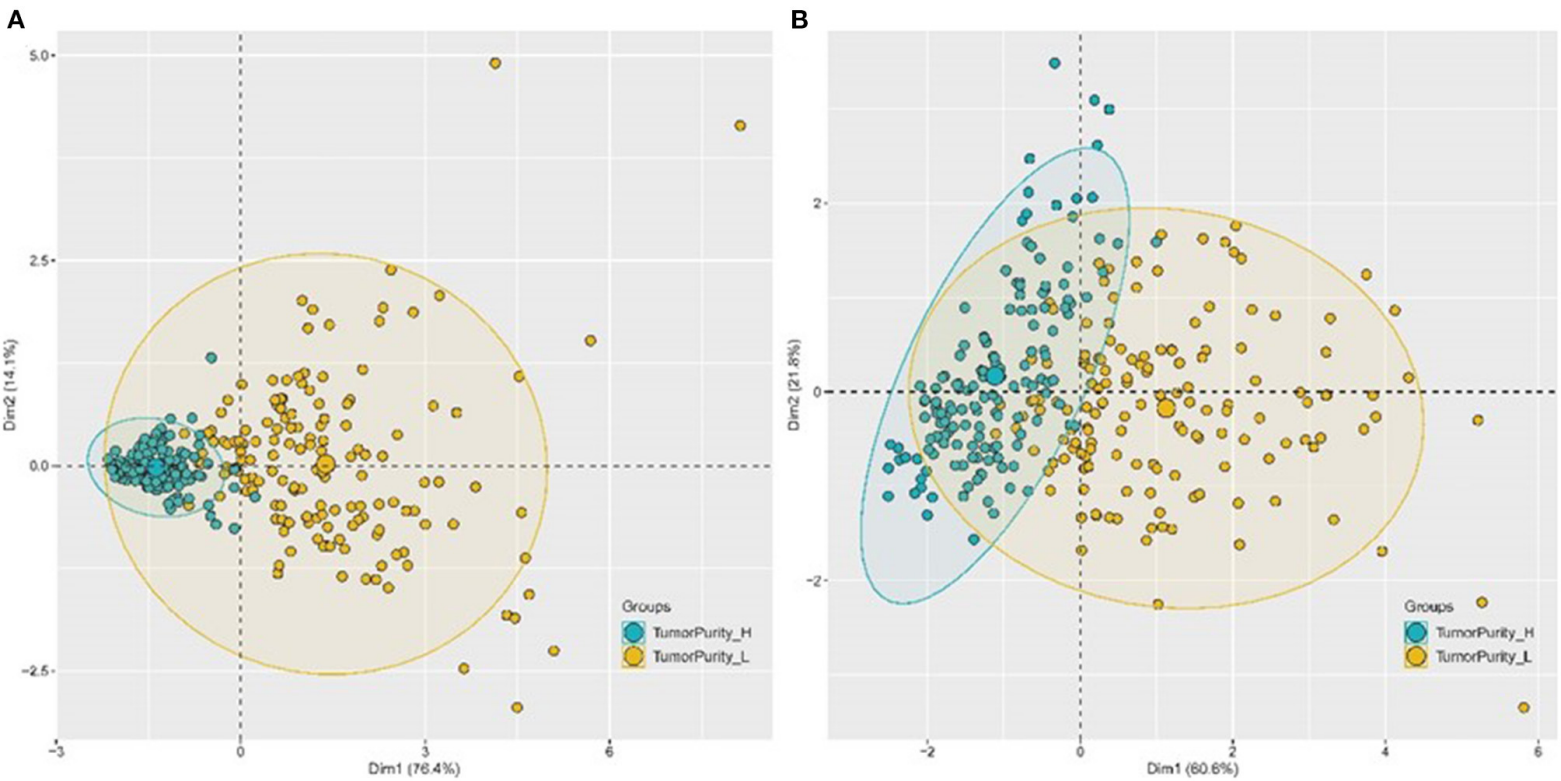
PCA chart of the classification effect of four genes on tumor purity. **(A)** PCA chart of the classification effect of four genes on tumor purity in TCGA database. **(B)** PCA chart of the classification effect of four genes on tumor purity in GEO database.

Four genes labeled tumor samples, and PCA analysis revealed that four genes could be better divided into high and low purity groups. The proportions of the first and second principal components of TCGA data and GEO data were 90.5 and 82.4%, respectively. The results indicated that the sample's tumor purity could be inferred through the expression of the four genes, and the gene expression and tumor purity were associated (Figure 15).

### LASSO Regression Analysis of Target Gene and Tumor Purity

We divided the 443 TCGA LUAD samples with complete clinical data into high and low groups according to tumor purity, and obtained CSGTPP (0.21^*^CCR2+ 0.06^*^GIMAP6+0.64^*^CD80+ 0.21^*^IL16) with LASSO regression analysis, and then divided the samples into high and low two groups. The simultaneous analysis revealed that the four genes were highly expressed in the high CSGTPP group (*P* < 0.0001) (Supplementary Figures 1A–D), and tumor purity was negatively correlated with CSGTPP (Supplementary Figure 2A). Using PCA, we determined the expression of four genes can be used to classify the two groups well with the first principal component and the second principal component value of 89.1% (Supplementary Figure 2B). Survival analysis revealed that patients in the high CSGTPP group exhibited a prognostic benefit (*P* < 0.0001), which echoed the poor prognosis of patients with higher tumor purity, as mentioned above (Supplementary Figure 2C). Finally, we explored the difference of CD274 gene expression in the CSGTPP two groups, and the results showed that the higher expression of CD274 in the high CSGTPP group (*P* < 0.0001) (Supplementary Figure 2D).

## Discussion

Recent advancement of omics technologies has significantly improved our understanding of the complexity and diversity of the immune components of the TME and its important impact on response to immunotherapy (9, 10). Further understanding of the tumor immune microenvironment will help improve the efficacy and response rates to immunotherapy. Therefore, an increasing number of studies have been conducted to study the TME and analyze immune cells' composition in tumor tissues. A large number of immune cells often gathered in and around tumors. There exist an inextricably link between these immune cells and tumor cells. Therefore, analyzing the composition and proportion of immune cells constitutes an integral part of studying the TME (11). Presently, there are two main methods for exploring the immune components of tumors. The first method includes high-precision single-cell-sequencing and single-cell-RNA-sequencing (scRNA-seq); the other method includes the speculation method that enables prediction based on the program using bulk RNA-seq data. However, estimation of tumor purity is considered crucial and warrants further investigation (12–14).

Recently, immunotherapy has emerged as a novel alternative therapeutic strategy for patients without driver genes mutation and has changed the treatment landscape of non-small cell lung cancer (NSCLC) (15, 16). Immunotherapy is an expensive therapy with significant clinical side effects; therefore, it remains critical to identify patients who will benefit from treatment with cancer immunotherapy. Therefore, it becomes increasingly essential to identify immune cells that exist in tumors; besides, deciphering immune cells present within the TME represents a significant area of implication in basic and clinical research.

Machine learning, an artificial intelligence method, utilizes complex algorithms in analyzing large-scale and heterogeneous data sets to extract useful patterns (17, 18). Machine learning has shown great potential with promising results in biomedicine, human genome project, cancer whole-genome project, international machine learning competition project, and other projects (19). Collection and analyses of large data sets related to medical treatment and patient prognosis can transform medicine into a data-driven and result-oriented discipline, which profoundly impacts disease detection diagnosis, prognosis, and response to therapy (20). We focused on the TCGA and GEO data of lung adenocarcinoma patients with high morbidity and high mortality rates globally to explore and mine the immune microenvironment and tumor purity in lung adenocarcinoma and identified some potential data-level relationships using existing research findings, based on the algorithms of regression trees, random forest regression, and random forest classification.

We used regression trees and random forest regression methods to analyze the predictive value of LUAD immune microenvironment for tumor purity and selected five immune microenvironment components that contributed the most to predicting tumor purity (CCR, T-helper-cells, Check-point, Treg, and TIL), which were negatively associated with tumor purity and tumor T, M and stage but not with N stage. A prognostic model was constructed based on 5 TIM genes, and 11 TIM-related genes were screened, which confirmed that immune risk characteristics were significantly related to the OS of LUAD patients. This relationship was still valid after controlling for clinicopathological characteristics. The risk score constructed based on 11 genes can be used as an independent prognostic factor for LUAD patients. We hypothesized whether tumor purity can be predicted at the gene expression level. Therefore, we investigated the predictive ability of 11 genes for tumor purity using random forest regression and random forest classification. Both TCGA and GEO data showed a good predictive value, and four genes (GIMAP6, CD80, IL16, CCR2) contributed the most to predicting tumor purity. GIMAP6, CD80, IL16, and CCR2 in LUAD were reported to be related to immune tolerance in NSCLC (21–24). CSGTTP was further estimated by LASSO regression and was found to be significantly associated with the patient's prognosis. The four genes' mRNA levels can clearly stratify high and low tumor purity and CSGTTP, confirming our hypothesis that these four genes could predict the tumor purity at the gene level. These four genes were derived from CCR, TIL, and checkpoint immune infiltration components; thereby, we further analyzed the association between TIM, gene, and tumor purity. In order to further explore whether tumor purity could be used as a predictive indicator of immunotherapy, we excavated that PD-L1 expression was positively correlated with CSGTTP, while tumor purity was negatively correlated with CSGTTP. PD-L1, as an effective indicator of immunotherapy, has been studied extensively. Although there are difficulties in estimating tumor purity, we can convert the estimation of tumor purity to the estimation of CSGTTP, thereby indirectly establishing the relationship between tumor purity and PD-L1. The higher CSGTTP indicates lower tumor purity, implying that patients may benefit from immunotherapy.

Currently, detection of PD-L1 expression remains the marker for identifying NSCLC patients that are more possibly respond to immunotherapy. Many chemoimmunotherapy trials have demonstrated the benefits of checkpoint inhibitors combined chemotherapy for LUAD (25) for all PD-L1 levels. Notably, patients with low PD-L1 expression exhibit poorer clinical outcomes, emphasizing PD-L1 as a biomarker even with chemotherapy as the first-line treatment. Although PD-L1 expression is important for predicting response, many trials highlighted that checkpoint inhibitor still cannot benefit from these drugs in sub-sets of patients with high PD-L1 expression. Tumor mutational burden (TMB) may be a potentially important biomarker for immunotherapy response, and the correlation between TMB and the response of immunotherapy has been demonstrated in a variety of tumor types (26). While limited by lack of testing platforms standardization and “high” TMB threshold, this indicator can only be applied to subsets of patients. The degree of lymphocyte infiltration observed in tumor tissue may have prognostic value. Previous studies have demonstrated high levels of tumor-infiltrating lymphocytes (TIL) with promising prognosis in NSCLC, which infiltrated more CD8 positive, CD3 positive, and CD4 positive TIL (27). High TIL density is believed to reflect the patient's tumor microenvironment, where T cells are inflamed. Therefore, the predictive value of TIL density as a biomarker of immunotherapy has also been studied. The five immune infiltration components we selected also included TIL. Immune gene expression characteristics represent a novel area of research on predictive indicators of immunotherapy. Studies have shown that it has potential use as a biomarker for anti-PD-1 and PD-L1 therapy, and may have predictive value, and is compatible with several cancer types and related to treatment response (28, 29).

The tumor immune score and matrix score can be obtained based on many algorithms to estimate tumor purity and the relationship between immune microenvironment and immunotherapy. Thus, the investigation of each component as the effect of immunotherapy remains an active area of research. However, thus far, tumor purity has not been thoroughly accessed as immunotherapy*-*associated markers. Tumor heterogeneity affects tumor immunotherapy, and tumor purity represents the homogeneity of tumors from a particular perspective, and it is easier to establish a relationship with the immune microenvironment, thereby guiding immunotherapeutic decisions. Although it remains challenging to estimate tumor purity accurately, the relationship between immune genes and tumor purity using the value of dominant genes on tumor purity is expected to infer tumor purity at the level of dominant genes and the correlation between tumor purity and immunotherapy. We believe that this study highlights the significance of tumor purity as an immunotherapy*-*related biomarker; however, further studies are warranted to validate these findings.

## Conclusion

In conclusion, CCR, T-helper-cells, Checkpoint, Treg, and TIL as the main immune infiltration components could better reflect tumor purity. We speculated that CSGTPP, derived from immune-related genes, could serve as a predictor of the response to immunotherapy and could stratify candidate for immunotherapy.

## Data Availability Statement

The datasets presented in this study can be found in online repositories. The names of the repository/repositories and accession number(s) can be found at: The Cancer Genome Atlas; GEO, GSE68465.

## Author Contributions

BL and HF have designed the research. HF and YZ analyzed data and wrote the paper. WY retrieved and collected data. CW and JL were responsible for drawing. BL revised the manuscript. All authors read and approved the final manuscript.

## Funding

This project was supported by Natural Science Foundation of Gansu Provincial China (No. 20JR10RA733).

## Conflict of Interest

The authors declare that the research was conducted in the absence of any commercial or financial relationships that could be construed as a potential conflict of interest.

## Publisher's Note

All claims expressed in this article are solely those of the authors and do not necessarily represent those of their affiliated organizations, or those of the publisher, the editors and the reviewers. Any product that may be evaluated in this article, or claim that may be made by its manufacturer, is not guaranteed or endorsed by the publisher.

## Supplementary Material

Supplementary Figure 1 Significant difference in screened genes expression in high and low comprehensive score of genes for tumor purity prediction (CSGTPP) group. **(A)** Significant difference of GIMAP6 in high and low CSGTPP groups; **(B)** Significant difference of CD80 in high and low CSGTPP groups; **(C)** Significant difference of CCR2 in high and low CSGTPP groups; **(D)** Significant difference of IL16 in high and low CSGTPP groups.

Supplementary Figure 2 Significant differences of tumor purity, sample classification, survival prognosis and CD274 expression in the high and low CSGTPP groups. **(A)** Significant difference in tumor purity between high and low CSGTPP groups; **(B)** PCA chart of the classification effect of four genes on tumor low and high CSGTPP in TCGA database; **(C)** Kaplan-Meier survival curves of the relative OS of high- and low-CSGTPP groups in TCGA database; **(D)** Significant difference of CD274 expression in the high and low CSGTPP groups.
