# Supplementary figures and images for: Identification of Signature Genes and Characterizations of Tumor Immune Microenvironment and Tumor Purity in Lung Adenocarcinoma Based on Machine Learning
Source: Front Med (Lausanne). 2022 Feb 25;9:843749. doi: 10.3389/fmed.2022.843749 (PMC8916235; doi:10.3389/fmed.2022.843749)

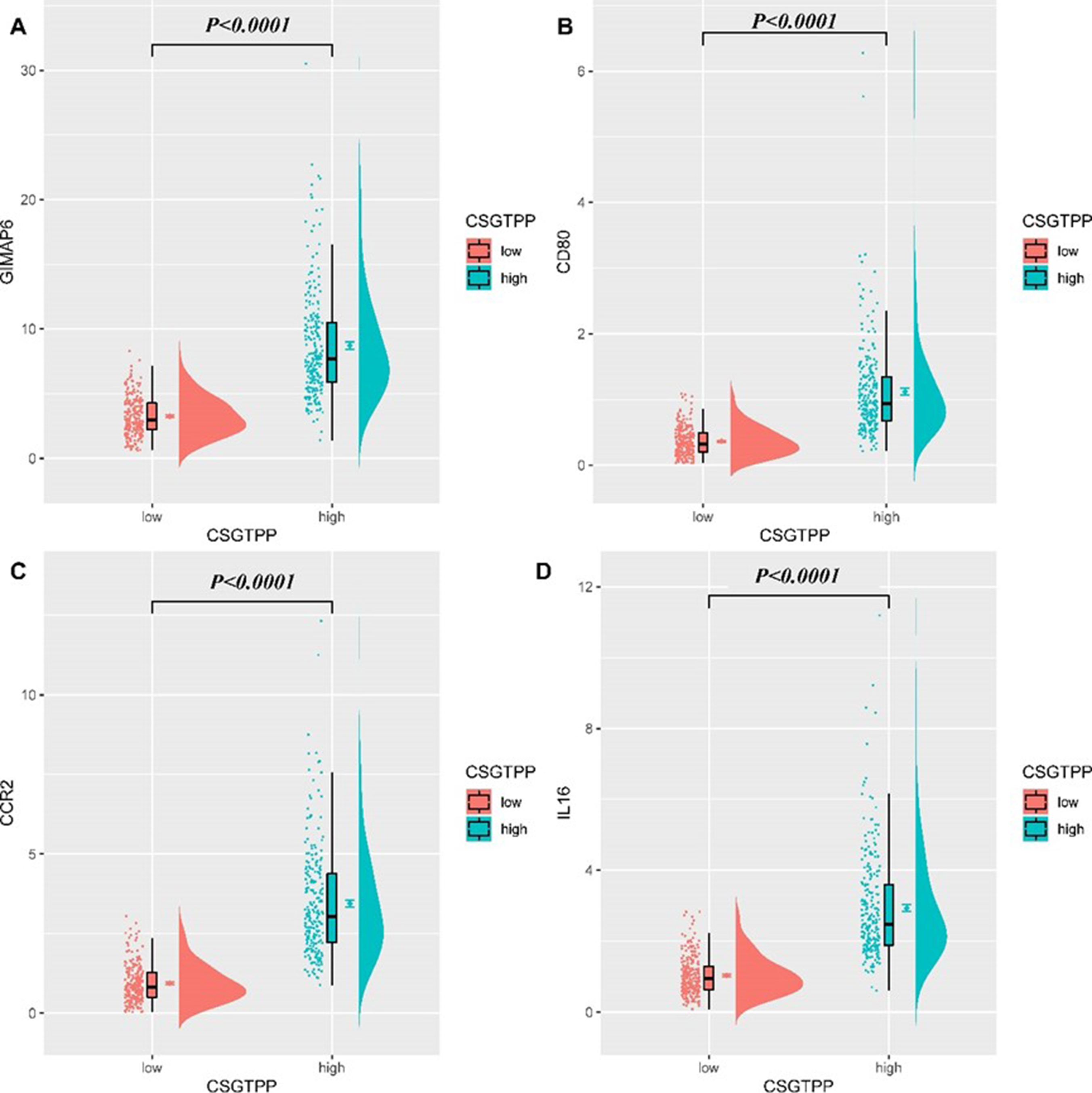

Supplement: Supplementary Figure 1 — Significant difference in screened genes expression in high and low comprehensive score of genes for tumor purity prediction (CSGTPP) group. (A) Significant difference of GIMAP6 in high and low CSGTPP groups; (B) Significant difference of CD80 in high and low CSGTPP groups; (C) Significant difference of CCR2 in high and low CSGTPP groups; (D) Significant difference of IL16 in high and low CSGTPP groups. [file Image_1.JPEG]

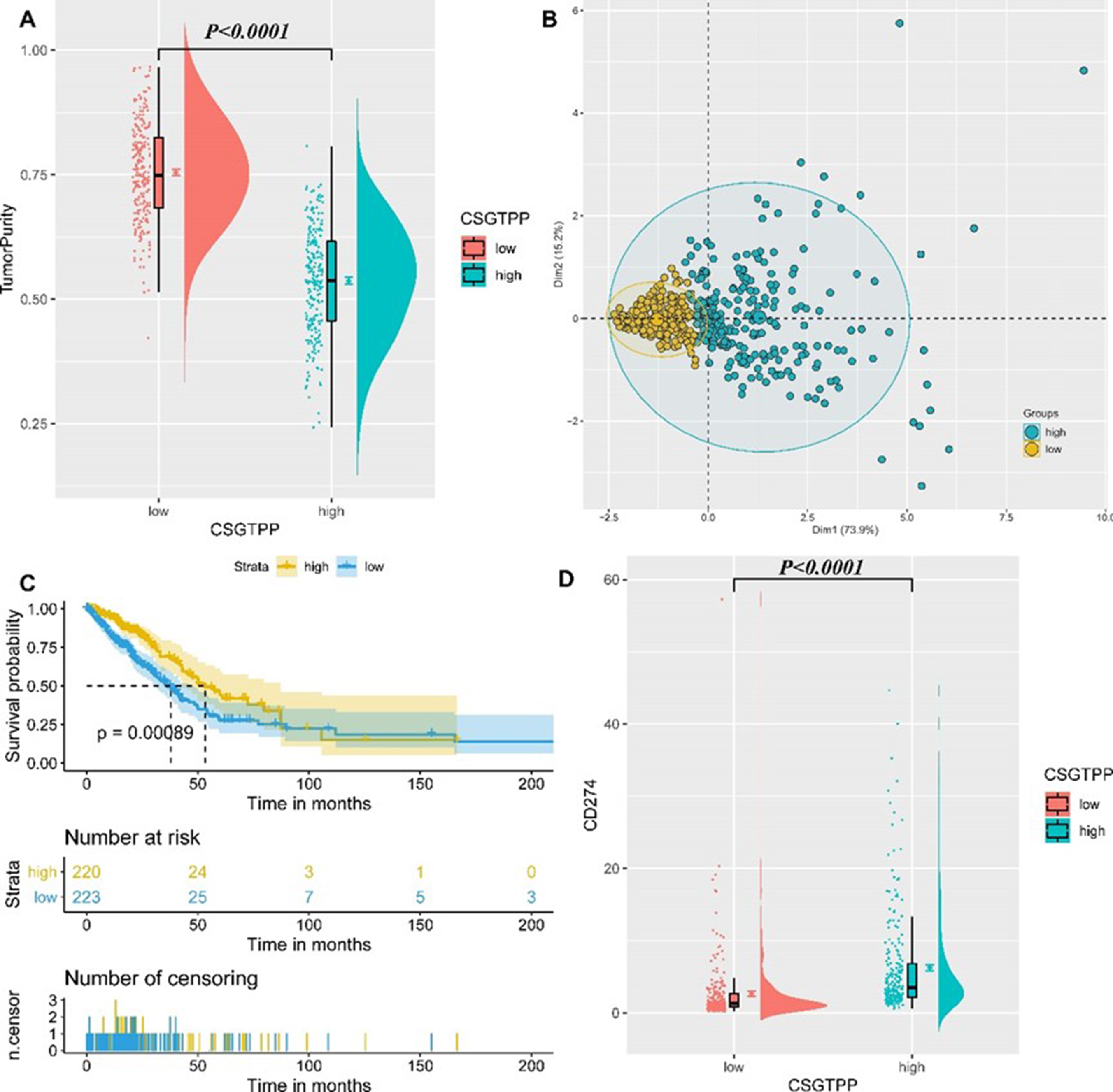

Supplement: Supplementary Figure 2 — Significant differences of tumor purity, sample classification, survival prognosis and CD274 expression in the high and low CSGTPP groups. (A) Significant difference in tumor purity between high and low CSGTPP groups; (B) PCA chart of the classification effect of four genes on tumor low and high CSGTPP in TCGA database; (C) Kaplan-Meier survival curves of the relative OS of high- and low-CSGTPP groups in TCGA database; (D) Significant difference of CD274 expression in the high and low CSGTPP groups. [file Image_2.JPEG]
